# Infectious Complications in a Patient Receiving Immunomodulatory Therapy for Gout

**DOI:** 10.7759/cureus.86555

**Published:** 2025-06-22

**Authors:** Sara Giddings, Meaghan Bethea, Chetna Hirani, Syed Ali Hussain

**Affiliations:** 1 Internal Medicine, Trinity Health Oakland Hospital, Pontiac, USA; 2 Rheumatology, Henry Ford Health, Detroit, USA

**Keywords:** cellcept (mycophenolate mofetil), drug-induced immunosuppression, gluteal abscess, krystexxa (pegloticase), medical comorbidities, mrsa bacteremia, refractory gout

## Abstract

Pegloticase is a recombinant uricase enzyme used in the treatment of refractory and severe tophaceous gout, often administered in combination with an immunomodulator such as mycophenolate mofetil and accompanied by pre-infusion medications, including corticosteroids and antihistamines to reduce hypersensitivity reactions. While this regimen is effective for managing gout, it may also increase the risk of opportunistic infections, particularly in patients with significant comorbidities. We present the case of a 75-year-old female patient with multiple comorbidities, including diabetes and chronic kidney disease, who developed a significant gluteal abscess that progressed to pelvic osteomyelitis and methicillin-resistant Staphylococcus aureus (MRSA) bacteremia while undergoing treatment for refractory gout with pegloticase (Krystexxa) and mycophenolate mofetil (CellCept). The patient has recovered from the infection; however, a 2.5 × 1.5 × 7.3 cm wound persists. The wound has remained clean with healthy granulation tissue and no exposed bone or signs of an active infection. This case highlights the importance of assessing infection risk in patients receiving immunomodulatory treatments for gout and demonstrates the need for close monitoring and multidisciplinary care, particularly in those with underlying comorbidities.

## Introduction

Gluteal abscesses are relatively common soft tissue infections; however, in immunocompromised individuals, they can progress to severe complications, such as bacteremia and osteomyelitis. In the case of an impaired immune response, these infections require prompt recognition and aggressive treatment. This report highlights the increased risk of deep-seated methicillin-resistant Staphylococcus aureus (MRSA) infection in a patient receiving biologic therapy with pegloticase (Krystexxa) and concurrent immunosuppressive treatment with mycophenolate mofetil (CellCept), raising concerns about infection risks associated with immunomodulatory regimens for gout.

MRSA infections typically present as skin or soft tissue infections but can also cause invasive disease, including osteomyelitis [1]. MRSA remains a significant cause of morbidity and mortality in immunocompromised patients, particularly those with chronic illnesses, diabetes mellitus, and immunosuppressive therapy [1,2]. In these patients, immune dysfunction impairs bacterial clearance, increasing the likelihood of systemic infection. This increasing susceptibility to invasive and recurrent MRSA infections often necessitates long-term antimicrobial therapy and surgical intervention [2]. Immunomodulatory therapies for gout management, such as pegloticase in combination with mycophenolate mofetil, may compromise host defenses and increase the risk of severe infections, including invasive MRSA disease.

In this report, we discuss a 75-year-old woman who presented with a left gluteal abscess complicated by pelvic osteomyelitis and MRSA bacteremia. This raised clinical concerns about the role of her immunomodulatory gout regimen, including pegloticase with pre-infusion corticosteroids and mycophenolate mofetil. The case emphasizes the importance of assessing infection risk when using dual immunomodulatory agents, particularly in individuals with additional risk factors, such as diabetes and chronic kidney disease.

## Case presentation

A 75-year-old female patient presented to the emergency department with ongoing pain in her left hip and posterior upper thigh. She had a past medical history of refractory gout, type 2 diabetes mellitus with peripheral neuropathy, chronic kidney disease stage III, atrial fibrillation on long-term anticoagulation, and uncomplicated bilateral hip arthroplasty in 2016 and 2018. Additionally, she had undergone surgery for a fractured femur in 2018 and had experienced limited mobility since, along with multiple self-reported falls. She also had a history of left lower extremity cellulitis and a chronic diabetic ulcer on her right great toe, which was culture-positive for Pseudomonas aeruginosa and MRSA without radiographic evidence of osteomyelitis. Both conditions were treated in the spring of 2024. Her refractory gout was managed with IV pegloticase (dose unknown), initiated in 2023, approximately one year before this admission. Three months before initiating the pegloticase infusions, the patient was started on prednisone 5 mg daily. Mycophenolate mofetil was subsequently added in 2024, five months before admission, as an immunosuppressive co-therapy at an initial dose of 500 mg twice daily. Table 1 summarizes some of the key clinical events. 

**Table 1:** Summarizes key clinical events in a chronological order MRSA: Methicillin-resistant Staphylococcus aureus.

Date	Clinical event
2018	Underwent femur fracture repair; developed long-term reduced mobility
Summer 2023	Initiated on low-dose prednisone (5 mg daily)
Fall 2023	Initiated on intravenous pegloticase for refractory gout
Spring 2024	Treated for left lower extremity cellulitis and diabetic ulcer on right great toe (cultures were positive for Pseudomonas aeruginosa and MRSA; no osteomyelitis)
Summer 2024	Started on mycophenolate mofetil as co-therapy with pegloticase
Fall 2024	Admitted for left gluteal abscess with MRSA bacteremia and suspected osteomyelitis

**Figure 1:**
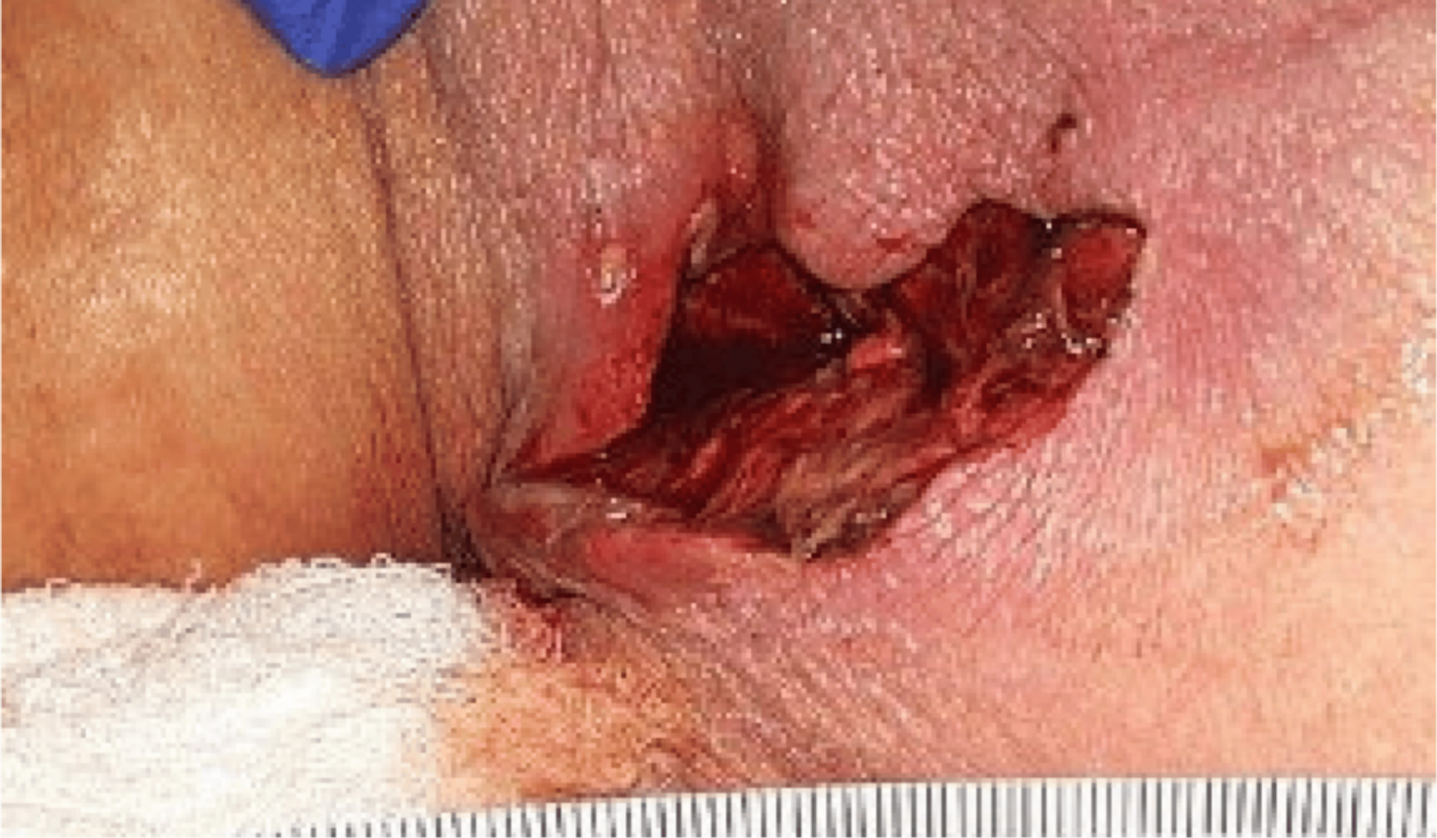
Post-debridement appearance of the initial left gluteal wound demonstrating an open cavity with exposed subcutaneous tissue and surrounding erythema

Seven days prior to admission, the patient reported rupture of a gluteal lesion with drainage of bloody, purulent fluid following a ground-level fall. She stated that the lesion had been present for approximately one year before this event. Upon examination, a left gluteal abscess cavity was noted with irregular wound margins, extending deep into the subcutaneous tissue. The surrounding skin appeared erythematous with signs of local inflammation. General surgery was consulted, and she subsequently underwent incision and drainage of the abscess, along with debridement (Figure 1). 

**Figure 2:**
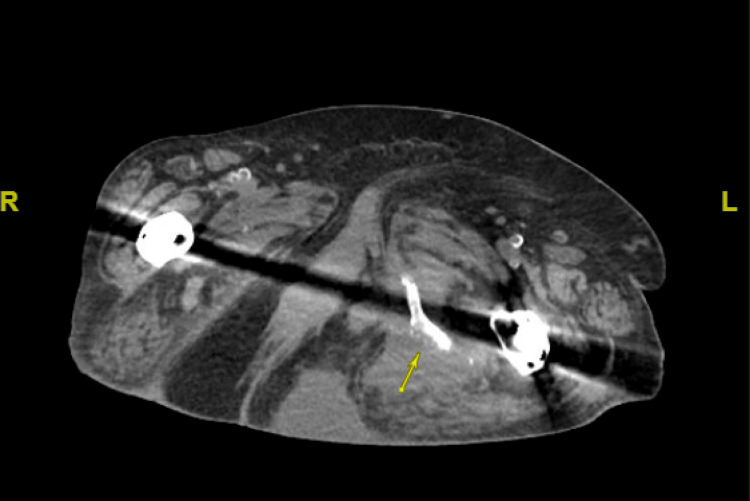
Axial non-contrast CT of the abdomen and pelvis Series 2, images #124–134, demonstrating a large complex fluid collection within the left gluteal and posterior thigh musculature (yellow arrow), measuring approximately 10 x 9 x 5.9 cm, with internal punctate calcifications. The collection extends inferiorly beyond the field of view and involves the left abductor muscles.

**Figure 3:**
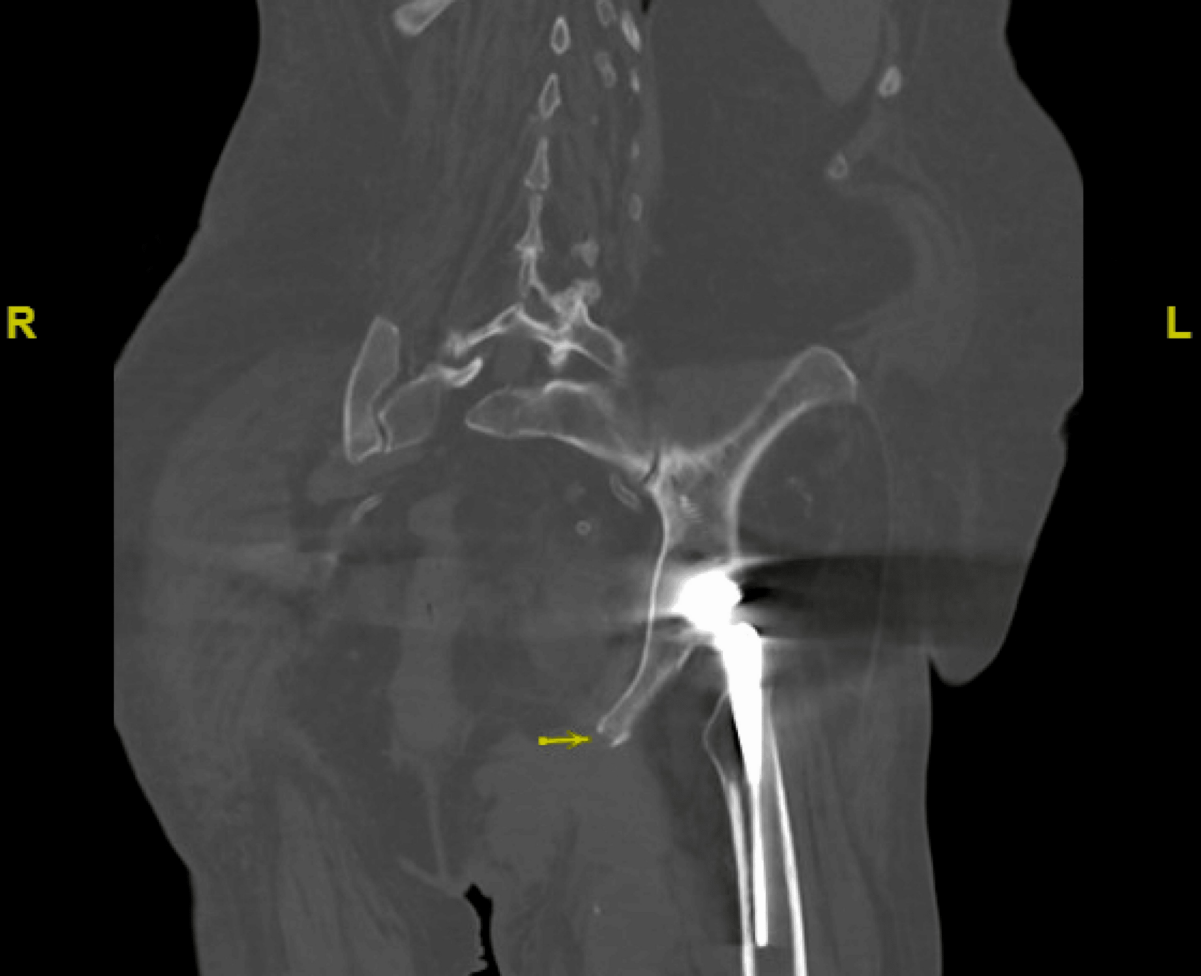
Coronal non-contrast CT of the abdomen and pelvis Series 4, images #99–106, showing cortical loss of the posterior left inferior pubic ramus with extension into the ischial tuberosity (yellow arrow), which was concerning for osteomyelitis.

**Figure 4:**
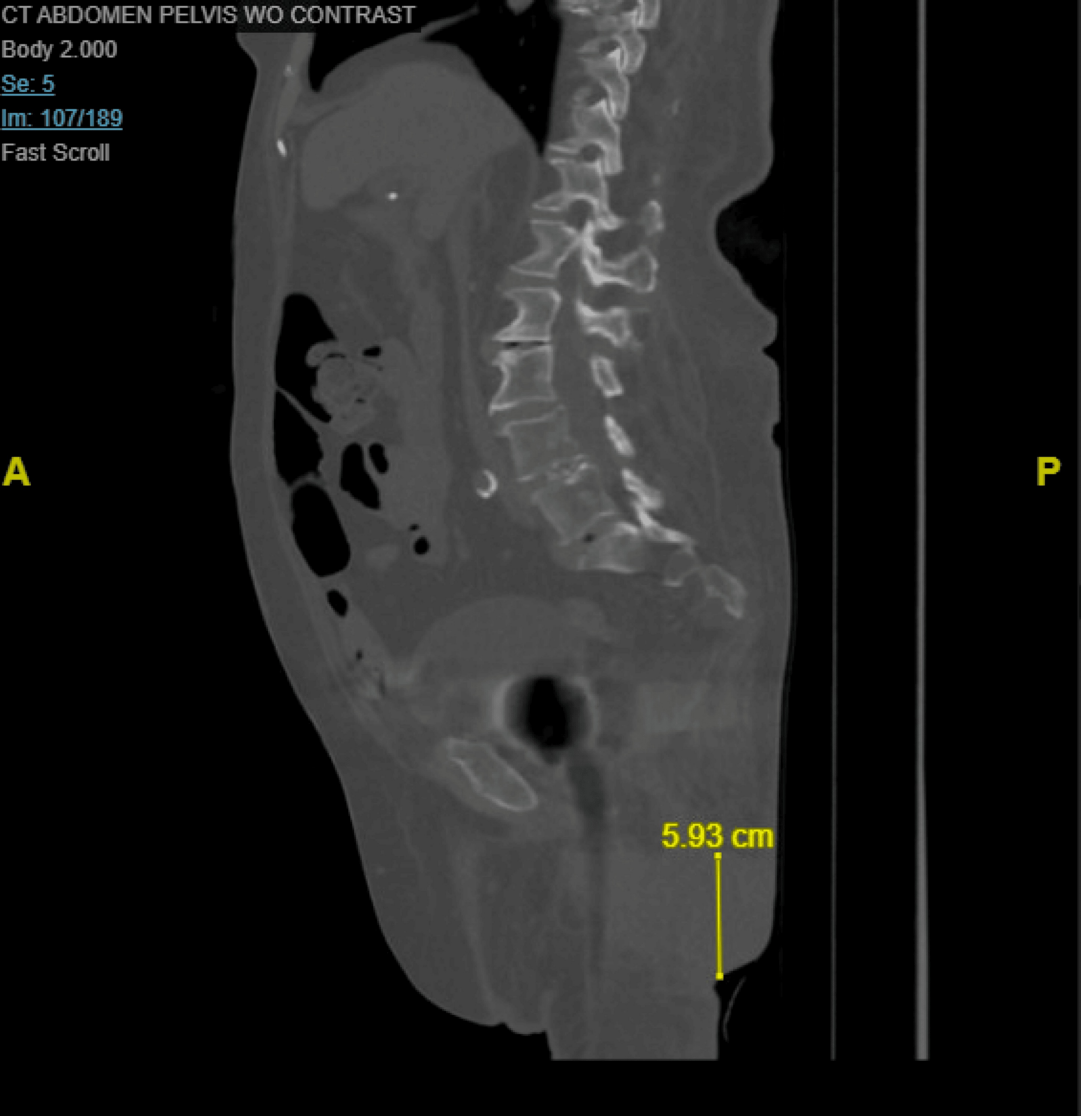
Sagittal non-contrast CT of the abdomen and pelvis Image demonstrating a fluid collection in the left gluteal region measuring approximately 5.93 cm in length, consistent with a gluteal abscess.

CT imaging of the abdomen and pelvis revealed large, lobulated fluid collections extending into the left abductor musculature, raising concerns for abscess formation. Additionally, bony erosions involving the left inferior pubic ramus and ischial tuberosity were noted, suggesting osteomyelitis (Figures 2-4).

The patient was initially started on intravenous vancomycin and cefepime. However, due to worsening renal function, vancomycin was discontinued and replaced with daptomycin. Wound and blood cultures subsequently grew MRSA, prompting discontinuation of cefepime. Notably, the patient was discharged 10 days prior due to acute-on-chronic anemia, and blood cultures at that time showed no growth. 

Due to the immunosuppressive effects of pegloticase and mycophenolate mofetil, both medications were discontinued during admission, and alternative treatment options were deferred to the patient’s rheumatologist. Given the severity of the MRSA infection, a transthoracic echocardiogram was obtained to evaluate for endocarditis; however, no vegetations were identified.

**Figure 5:**
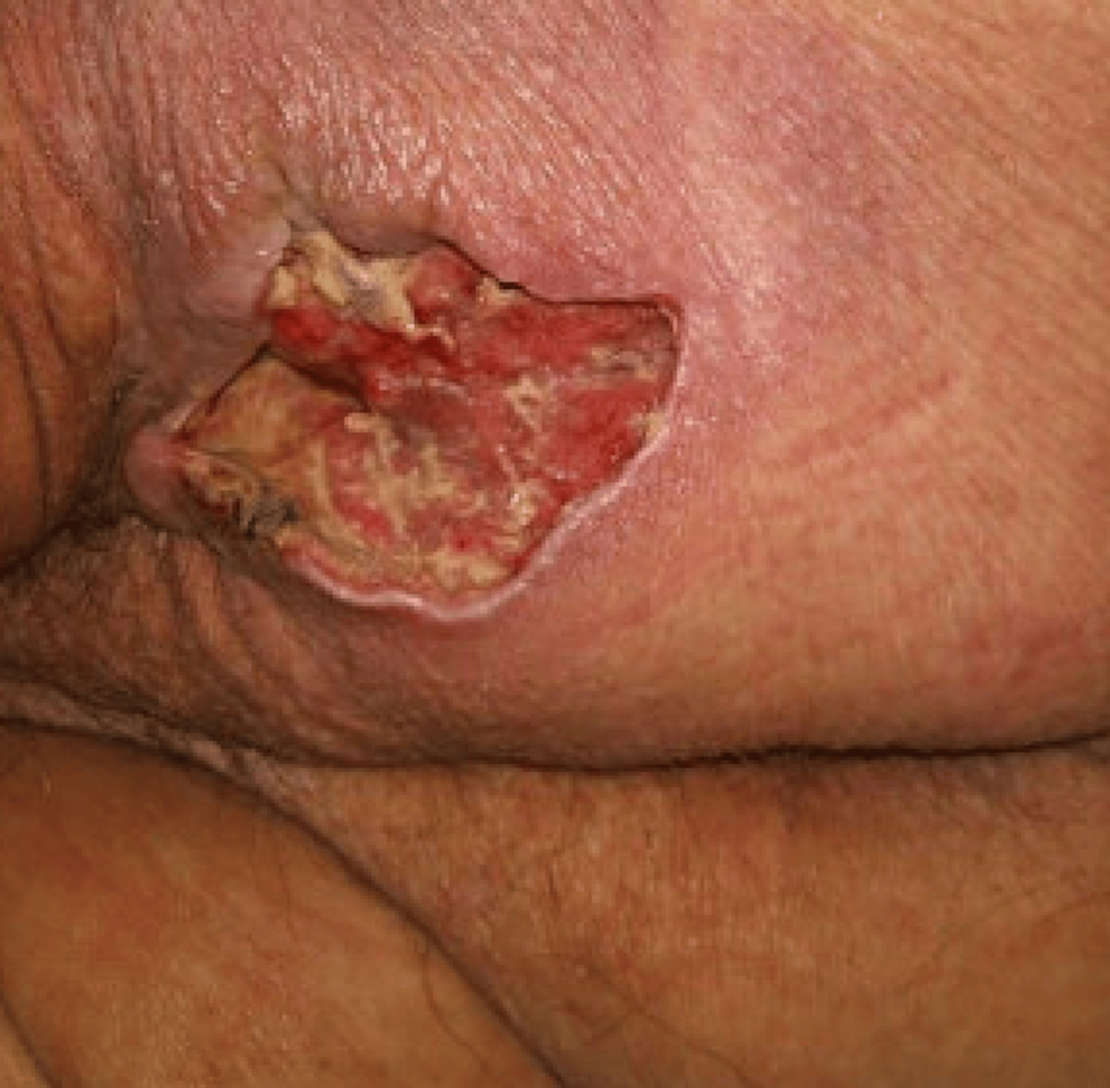
Wound with clearly defined margins and exposed subcutaneous tissue Wound observed eight days post-debridement and one day before discharge. The wound bed demonstrates early healing with fused tissue surfaces and interlacing yellow streaks, likely representing slough or fibrin deposition.

The patient underwent daily wound care and dressing changes throughout her 11-day hospital stay. Figure 5 illustrates the progression of the wound from the initial stage shown in Figure 1 up to one day before discharge.

She was subsequently discharged to a subacute rehabilitation facility to complete an eight-week course of intravenous daptomycin administered via a Groshong catheter, given her underlying chronic kidney disease. She was advised to follow up with the surgical team for continued wound care management and nephrology to monitor kidney function and creatine kinase levels while on daptomycin. At discharge, pegloticase remained discontinued, and the patient was maintained on a reduced dose of mycophenolate mofetil and daily prednisone.

**Figure 6:**
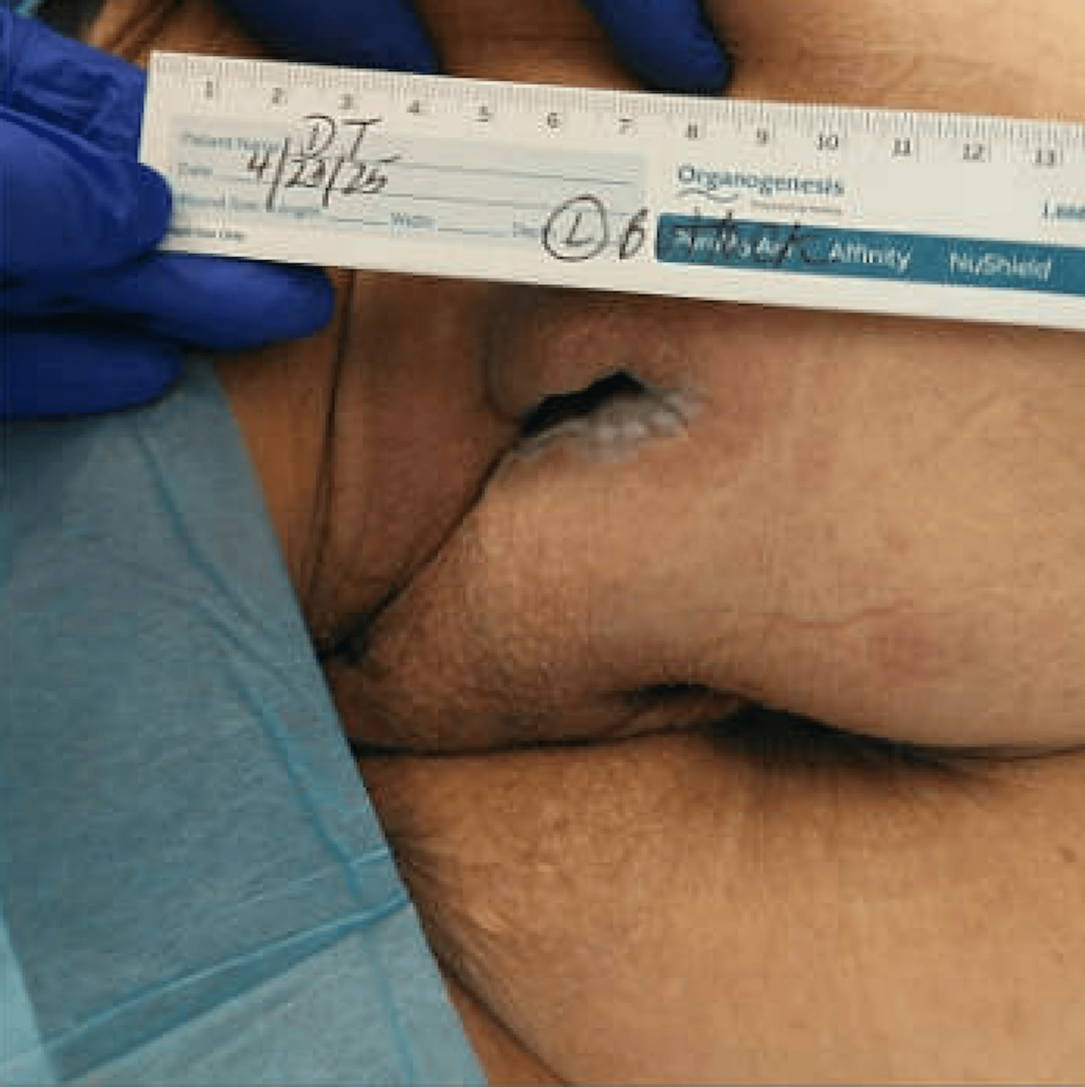
Recent image of the left gluteal wound, taken five months post-discharge The wound exhibits well-healed, epithelialized margins and remains partially open, with no visible signs of inflammation or infection.

At the six-month follow-up, the patient had undergone multiple outpatient surgical visits. A wound vacuum-assisted closure (VAC) device was initially placed but later discontinued due to frequent dislodgement and dressing complications related to diarrhea. After resolution of the diarrhea, negative pressure wound therapy was successfully reinitiated. The wound remained over the left ischial buttock and extended to the ischial tuberosity, demonstrating improvement. At present, the ulcer shows healthy granulation tissue at the base with normal-appearing surrounding skin (Figure 6). 

Despite a referral to plastic surgery for possible flap reconstruction, the patient remained hesitant to proceed due to concerns about the postoperative recovery course.

Her advanced chronic kidney disease, type 2 diabetes with peripheral neuropathy, and limited mobility following orthopedic surgery likely predisposed her to wound development and impaired healing. The origin of the wound remains unclear, as the patient reported a raised lesion in the gluteal region for the past year. In combination with these risk factors, the use of immunomodulatory agents may have further compromised her immune defenses, contributing to the progression and severity of the soft tissue infection.

## Discussion

This case illustrates the unique, infectious risks associated with using pegloticase in conjunction with immunomodulators, such as mycophenolate mofetil, and corticosteroids in the management of chronic, tophaceous gout. While this combination enhances pegloticase efficacy by mitigating immunogenicity [3], it also suppresses the immune system, thereby increasing vulnerability to infections like MRSA. This is exemplified by the patient's severe gluteal abscess and subsequent MRSA bacteremia and osteomyelitis. 

Pegloticase transforms uric acid into allantoin, a more soluble compound easily eliminated by the kidneys, thus promoting its excretion [4]. Pegloticase, a recombinant uricase enzyme, is reserved for chronic refractory or tophaceous gout when conventional urate-lowering therapies have failed. Despite its efficacy, pegloticase is highly immunogenic, and up to 89% of patients develop anti-drug antibodies during treatment, which reduces drug efficacy and increases the risk of infusion reactions [5]. Co-administration with immunosuppressants, such as mycophenolate mofetil, has been shown to improve treatment response by reducing the formation of antibodies [3].

However, mycophenolate mofetil, a purine synthesis inhibitor, broadly suppresses T- and B-lymphocyte proliferation, diminishing cell-mediated and humoral immune defenses [6]. When combined with systemic corticosteroids, which impair neutrophil function, macrophage activity, and cytokine production [7], patients face a compounded risk of infection. This immunosuppressive triad may significantly compromise the host's ability to contain and clear bacterial pathogens like MRSA.

MRSA remains a formidable cause of bacteremia, particularly in hospitalized or immunocompromised individuals, and has a well-documented capacity to spread to distant sites, such as bones, joints, and soft tissue [1,2]. This was demonstrated in our patient, who had a deep gluteal abscess, along with radiographic signs of pelvic osteomyelitis and subsequent wound and blood cultures that confirmed an MRSA infection. The patient's comorbid type 2 diabetes further heightened infection risk by impairing neutrophil chemotaxis and phagocytosis, while chronic kidney disease stage III introduced limitations in antimicrobial clearance and immune regulation [8]. From a management perspective, the patient's renal impairment further complicated care. Vancomycin, often the first-line agent for MRSA, was initially used but later replaced with daptomycin due to worsening renal function. Daptomycin is bactericidal and effective against MRSA, with a relatively favorable renal safety profile, making it a valuable alternative in such scenarios [9]. However, it requires monitoring for myopathy and rhabdomyolysis, particularly in elderly or renally impaired patients.

This case also raises broader questions about the safety and appropriateness of aggressive immunomodulatory therapy for gout in elderly patients with multiple comorbidities. Although mycophenolate mofetil can help minimize the immunogenicity of pegloticase and improve its effectiveness, its application must be weighed carefully against the potential risk of infection, especially in individuals with existing immunosuppression or compromised kidney function. Given that gout is a chronic, non-life-threatening condition, it may be prudent to consider alternative approaches or more intensive infectious disease monitoring in such high-risk individuals. Pegloticase is typically reserved as a last-line therapy for chronic gout when initial agents such as allopurinol or febuxostat are ineffective or poorly tolerated; therefore, therapeutic alternatives may be limited in the event of complications related to pegloticase. However, it may be considered as a short-term bridging treatment to rapidly lower uric acid levels, particularly in situations where prolonged immunosuppression is undesirable due to high infection risk, before reintroducing standard therapies [10]. Additionally, in patients who are unable to tolerate allopurinol or febuxostat and do not have significant renal impairment, probenecid, a uricosuric agent, may be a reasonable option before progressing to pegloticase.

Additionally, guidelines for infection screening before initiating dual immunomodulatory therapy in gout patients are currently lacking. This case supports the need for pre-treatment risk stratification tools, a baseline infectious disease evaluation, and potentially antimicrobial prophylaxis or decolonization protocols for selected patients.

Thus, the key clinical takeaways from this case study are: 1) Immunomodulatory co-therapy (e.g., mycophenolate mofetil) may enhance pegloticase efficacy but increases the risk of infection, especially in patients with immune-compromising comorbidities; 2) All conventional chronic gout therapies should be optimized before initiating pegloticase, and a careful risk-benefit assessment is critical in patients with multiple comorbidities; 3) Close monitoring is essential for patients receiving biologics or immunosuppressants, particularly those with limited mobility (e.g., a sedentary lifestyle with consequent skin breakdown) or increased wound susceptibility (e.g., diabetes, peripheral vascular disease, tobacco use); and 4) A multidisciplinary approach involving relevant specialists and regular follow-up is recommended when managing patients on immunomodulatory regimens.

## Conclusions

This case illustrates the potential infectious complications associated with dual immunomodulatory therapy in patients with gout. The combination of pegloticase with pre-infusion steroids and mycophenolate mofetil likely increased susceptibility to serious infections, including abscesses, MRSA bacteremia, and osteomyelitis. Clinicians should remain vigilant about the risks associated with these therapies and consider referring high-risk patients for early consultation with infectious disease specialists. Future research is needed to better understand the relationship between pegloticase, immunosuppression, and the development of opportunistic infections. Specifically, clinical studies incorporating infection risk stratification in pegloticase-treated patients could guide the development of targeted risk assessment and preventive strategies for vulnerable populations.
